# Syndecan-1 in Cancer: Implications for Cell Signaling, Differentiation, and Prognostication

**DOI:** 10.1155/2015/796052

**Published:** 2015-09-01

**Authors:** Tünde Szatmári, Rita Ötvös, Anders Hjerpe, Katalin Dobra

**Affiliations:** Division of Pathology, Department of Laboratory Medicine, Karolinska Institutet, Karolinska University Hospital, F-46, 141 86 Stockholm, Sweden

## Abstract

Syndecan-1, a cell surface heparan sulfate proteoglycan, is critically involved in the differentiation and prognosis of various tumors. In this review, we highlight the synthesis, cellular interactions, and the signalling pathways regulated by syndecan-1. The basal syndecan-1 level is also crucial for understanding the sequential changes involving malignant transformation, tumor progression, and advanced or disseminated cancer stages. Moreover, we focus on the cellular localization of this proteoglycan as cell membrane anchored and/or shed, soluble syndecan-1 with stromal or nuclear accumulation and how this may carry different, highly tissue specific prognostic information for individual tumor types.

## 1. Introduction

The syndecan family consists of four transmembrane heparan sulfate proteoglycans (HSPGs) mainly present on the cell surface [1, 2]. The structures of these different syndecans show high homology in vertebrates and invertebrates [3, 4]. All four syndecans are built up of a core protein decorated with varying number of glycosaminoglycan (GAG) side chains. Syndecans exert their functions mainly through these GAG chains, but the different domains of the core protein have distinct roles as well [5, 6]. Syndecan-1 and syndecan-3 carry both heparan sulfate (HS) and chondroitin sulfate (CS) chains, whereas syndecan-2 and syndecan-4 carry only HS chains [7].

Syndecan-1 is the main syndecan on the basolateral surface of epithelial cells in adult tissues, it is transiently expressed by mesenchymal cells during development, and it is also found in distinct stages of differentiation of lymphoid cells [1]. Syndecan-2 is present primarily on cells of mesenchymal origin [8], syndecan-3 is primarily expressed by neuronal tissue and cartilage [9, 10], and syndecan-4 is ubiquitously found in most tissues [11, 12]. Syndecans are involved in a wide range of biological processes including growth and differentiation [13], cell spreading, cell adhesion [5], cell migration, cytoskeletal organization [14–16], infiltration, and angiogenesis [6, 17].

## 2. The Structure of Syndecan-1 and the Biosynthesis of Heparan Sulfate Chains

The gene encoding for syndecan-1 consists of five exons and is located in human chromosome 2; the first exon encodes a signal peptide; the second exon encodes the attachment sites for heparan sulfate; the third and fourth exons encode the site of chondroitin sulfate binding site and the fifth exon encodes transmembrane and cytoplasmic domains. The expression of syndecan-1 depends largely on the tissue type and on the developmental stage. The synthesis of syndecan-1 occurs in the early stages of differentiation [18, 19].

Structurally, syndecan-1 is composed of a 310 amino acids long core protein, which consists of an extracellular domain with GAG side chains, a transmembrane domain, and a highly conserved cytoplasmic domain [2]. The synthesis of the polypeptide chain of the core protein begins on membrane-bound ribosomes and continues in the lumen of the endoplasmatic reticulum. HS biosynthesis occurs in the Golgi apparatus and involves the participation of several enzymes that catalyze the elongation of the disaccharides [20]. The formed polysaccharide chains are further modified by epimerization, deacetylation, and addition of sulfate groups at different positions by the action of several other enzymes such as epimerases and sulfotransferases. Finally, the PGs are delivered by exocytosis to the cell surface [21, 22].

The GAG chains are covalently attached to the core protein in the syndecan-1 via common linkage tetrasaccharides: a serine on the protein core is linked by xylosyl transferases to a xylose on the GAG chain, which is in sequence attached to two galactose residues and one glucuronic acid residue. Acidic amino acids surrounding these Ser-Gly repeats promote substitution with HS as well, possibly by helping the first N-acetylglucosamine (GlcNAc) transferase to act on the linkage sequence [23, 24].

HS consists of repeating disaccharide units of N-acetylglucosamine (GlcNAc) with glucuronic acid (GlcA) or GlcNAc with iduronic acid (IdoA), whereas chondroitin sulfate is composed of disaccharide units of N-acetylgalactosamine (GalNAc) and GlcA [23, 25]. This implicates 50–200 negatively charged disaccharide units in each GAG chain due to the attached sulfate groups [26] which can bind a large number of positively charged molecules. Moreover, because of this negative charge, GAG chains are pushed from each other and expand into extracellular space to increase the area of their interaction [27].

Modulation of syndecan-1 initiates a significant alteration in the expression of enzymes involved in HS biosynthesis, metabolism, and turnover, particularly SULFs [28], the enzymes responsible for selective removal of 6-O sulfate groups from HS chains. Since the ability of syndecan-1 to bind growth factors and initiate signaling is dependent on the amount, position, and the orientation of the sulfate groups on the HS chains [29–34], the modulation of these enzymes by syndecan-1 might represent an important feedback mechanism. Experimental data also suggest that syndecan-1 coordinates the expression of various proteoglycans in different tumor types, although the effect varies largely from one tissue type to other [32, 35, 36]. These alterations might lead to modifications of the HS pool of the cells, ultimately modulating the effects of syndecan-1 on signaling.

### 2.1. The Role of Glycosaminoglycan Chains

GAG chains bind various protein ligands in a structure-dependent manner, but depending on the core protein, their position will be different. The ligand binding to proteoglycans is extremely complex because proteoglycans carry multiple GAG chains that may function cooperatively. Furthermore, cooperation between the core protein and attached GAG chains may also occur. Syndecan-1 exerts its functions predominantly through its HS chains, by binding various morphogens and growth factors with varying affinity to high and low sulfated regions [37, 38]. The extent to which HS is sulfated can vary depending on tissues and cell type, which also has consequences for the functionality of HSPGs in any given tissue. Ligand binding to mature HS is also affected at the cell surface by the two sulfatases (SULF-1 and SULF-2) and heparanases [20].

Syndecan-1 acts as a coreceptor by simultaneously binding various growth factors such as fibroblast growth factors (FGFs), vascular endothelial growth factor (VEGF), Wnt, hepatocyte growth factor (HGF), and their receptors through its HS chains [17, 39–41], thereby stabilizing the growth factor/growth factor receptor complexes. This is followed by activation of downstream tyrosine kinase pathways. Syndecan-1 facilitates the FGF2-FGFR1 complex formation in different tumor types, comprising lymphomas [42], breast cancer [43], and prostate cancer [29]. It also promotes HGF-induced signaling in myeloma through its receptor MET and downstream activation of Ras/MAPK and PI3/Akt signaling pathways, resulting in enhanced cell proliferation and survival [30]. WNT1 signaling and tumor growth are enhanced by syndecan-1 in mammary gland tumors [39], and syndecan-1 has a role in the ability of Wnt1 to induce the accumulation of mammary progenitor cells [31]. We have recently shown that syndecan-1 influences multiple signaling pathways in malignant mesothelioma, a highly aggressive mesenchymal tumor. Several growth factors (epithelial growth factor (EGF), platelet-derived growth factor (PDGF), and FGF) and their receptors were finely tuned by syndecan-1. Moreover these effects go beyond the capacity of syndecan-1 to bind cell-surface receptors, as the expression of downstream effectors was also influenced, often at much higher extent than the syndecan-1 itself, involving ERK/MAPK, Akt, and p38/MAPK signaling cascades and MYC, JNK, JUN, and ETS-1 expression as downstream transcription factors [32]. The delicate control of multiple signaling pathways regulated by syndecan-1 might imply feedback loops and/or epigenetic regulatory mechanisms that collectively affect gene transcription. In contrast, ETS-1 and syndecan-1 are inversely correlated in colon carcinoma [33], pointing toward the cell type specificity of the effect of syndecan-1.

Considerable attention has been focused also on interactions between syndecan HS chains and numerous bioactive molecules such as chemokines and other extracellular matrix (ECM) components [44]. GAG chains interact with chemokines such as CCL2 (MCP-1), CCL5 (RANTES), CXCL12 (SDF-1), and induces chemotaxis of various cells in a chain length- and sulfation pattern-dependent manner [45, 46].

### 2.2. The Role of the Core Protein

The first evidence that the syndecan-1 core protein has biological function came from studies on mouse mammary tumor cells, where a truncated mutant of syndecan-1 lacking both the transmembrane and cytoplasmic domains was shown to be secreted into the culture medium as soluble glycanated syndecan ectodomain [47].

The HS chains on syndecan-1 are not always required for the initiation of a signaling event, especially for cytoskeletal signaling. Recent discoveries indicate that syndecan-1 core proteins also have biological functions and can modulate cell behavior independent of HS. These regulatory sequences have been proposed to act with both autocrine and paracrine mechanism and could well represent novel targets for therapeutic interventions, particularly in diseases such as cancer [48].

#### 2.2.1. Extracellular Domain

The syndecan-1 core protein binds via the extracellular domain to both *α*v*β*3 and *α*v*β*5 integrin during angiogenesis or with *α*v*β*1 integrin during reepithelialization of lung tissue, thus potentially forming ternary complexes between extracellular molecules, a cell surface receptor, and a PG component, similar to the ones well recognized for growth factors, their corresponding receptors, and HS chains [6, 49–51]. Similarly, syndecan-1 activates the *α*v*β*3 integrin by binding the insulin-like growth factor-1 receptor (IGF1-R) and integrins directly via its ectodomain, assembling in a ternary receptor complex. It was demonstrated that activation of integrin does not require the other regions of syndecan-1 core protein (cytoplasmic or transmembrane domain) or the HS chains or their attachment sites [5, 52]. Since normal epithelial cells do not express these integrins but in most carcinomas they are upregulated, the syndecan-1-coupled ternary receptor complex is present mostly in tumors. This association between syndecan-1 and *α*v*β*3 and *α*v*β*5 integrins was described for carcinomas [52, 53], myeloma [17], fibroblasts [54], and activated vascular endothelial cells [55].

Syndecans can trigger signaling leading to cell adhesion and spreading either by exposing binding sites on fibronectin for *β*1 integrin engagement or by modulating the activation state of the *β*1 integrin [56].

#### 2.2.2. Transmembrane and Cytoplasmic Domains

The transmembrane and cytoplasmic domains of syndecans do not have intrinsic kinase or catalytic activity by themselves and, however, by multimerization or interaction with different intracellular components like GTPases or kinases play an important role in propagating the signal transduction [57]. The conserved GGLVG transmembrane domain of syndecan-1 mediates dimerization [58, 59]. Usually, this occurs in lipid rafts, the parts of the plasma membranes containing combinations of glycosphingolipids and cholesterol [60]. Lipid rafts are essential for receptor binding and signal transduction from the cell surface into the cell. The conserved motif GGLVG is also necessary for retaining cholesterol in the membrane [58]. Decreasing the cholesterol level in the lipid rafts leads to their degradation and to the damage of the signaling cascade [61].

The short cytoplasmic domain of syndecan-1 interacts with a number of cytosolic proteins and plays a role in endocytosis. It has two conserved C1 and C2 sites that flank the variable region V. The conserved C1 site mediates syndecan dimerization and interacts with numerous intracellular proteins such as ezrin, tubulin, and cortactin that regulate the organization of the cytoskeleton [62, 63]. The conserved C2 domain (EFYA) binds to PDZ-binding proteins, such as synbindin, synectin, CASK [64], CASK/LIN-2, and syntenin that play an important role in vesicular transportation, adhesion, synaptic signaling, neuronal migration, and metastasis formation [65–67]. The cytoplasmic domain of syndecan-1 also interacts with *α*_6_ *β*_4_ integrin and regulates activation of ErbB2 by the integrin [68].

## 3. Shedding of the Extracellular Domain and Its Role in Signaling

Syndecans can be found in two forms: membrane-incorporated and soluble. The soluble form is the ectodomain containing GAG chains that have been shed from the cell surface. The proteolysis of syndecan-1 occurs at a specific juxtamembrane cleavage site between amino acids A243 and S244 [69] and is performed by a number of matrix metalloproteases: MMP7 [49], MMP9 [70], MMP14 [71], the membrane-associated MT-MMP1, ADAM10, and ADAM17, and other sheddases.

The shedding is regulated by a large number of extracellular stimulating agents: growth factors, chemokines, trypsin, heparanase, bacterial virulence factors, insulin, and cellular stress [72, 73]. Among growth factors, FGF-2 was shown to activate MMP-7 mediated shedding [74]. Heparanase accelerates MMP-9 mediated shedding of syndecan-1 in both myeloma and breast cancer [17]. The heparanase mediated syndecan-1 shedding occurs through upregulation of ERK phosphorylation that leads to enhanced expression of MMP-9 [75, 76]. Recent studies also show that heparanase induced shedding stimulates the expression of the active protease and through this stimulates tumor growth and spreading [75, 77]. Even though syndecan-1 shedding can occur constitutively, shedding is induced and accelerated in tumors, following the activation of both G-protein coupled receptors and protein tyrosine kinases by specific agonists, including thrombin and epidermal growth factor [78]. Certain signal transducers, such as protein kinase C and nuclear transcription factor NF-kB, also influence the shedding of ectodomain [26]. Interestingly, syndecan-1 can itself participate in regulation of metalloproteinases as the HS chains on the core protein suppresses the shedding [73]. In addition, interaction of cytoplasmic domain with Rab5 affects shedding of ectodomain [79] and the phosphorylation of tyrosines in the conserved sites of the cytoplasmic domain initiates the shedding of the ectodomain [80, 81].

Chemotherapy can also induce shedding of syndecan-1 in malignant myeloma, predominantly via ADAMs, and this shed syndecan-1 is functionally active, leading to relapse and formation of a more aggressive phenotype [82]. On the other hand, in colorectal cancer shed syndecan-1 induces resistance to chemotherapy via the EGFR pathway [83].

The shedding of syndecan-1 has biological significance, as the shed ectodomain contains the intact HS chains, retaining its ability to bind growth factors and other ECM components, creating a chemotactic gradient. Shed ectodomain can also compete with the membrane-bound syndecan-1 for ligand binding, and it can sequester the HS binding factors in the extracellular matrix and thereby modulate their biological functions [84]. Moreover, shed syndecan-1 is able to deliver growth factors to other cells, as it was shown for syndecan-1 originating from multiple myeloma cells, which is released in the medium and is taken up by the surrounding stromal cells [85]. As a consequence, membrane-bound and soluble syndecan-1 can have opposite effects on cancer cells and can influence a wide range of behaviors such as tumor growth and metastasis, chemokine localization, leukocyte trafficking, and pathogen virulence. Thus, the change in localization of syndecan-1 from the cell surface to the extracellular matrix has distinct and important pathological effects. This was experimentally verified by a number of studies. For example, in breast cancer overexpression of wild type syndecan-1 increased proliferation, but overexpression of constitutively shed syndecan-1 inhibited it [86]. Heparanase mediated shedding of syndecan-1 correlates with enhancement of both VEGF [17] and HGF [87] signaling and affects angiogenesis. It was also shown that shed syndecan-1 and predominantly its HS chains from stromal fibroblasts were required for breast carcinoma angiogenesis [88] and growth of breast cancer cells was stimulated by shed syndecan-1 via activation of FGF-2 [41].

Taken together, syndecan-1 shedding is an important phenomenon as significant levels of syndecan-1 are shed by tumors. This can be used not only as biomarkers for monitoring disease progression and treatment response but also when considering strategies to improve the effect of targeted cancer therapy, by inhibiting metalloproteases or heparanase.

## 4. The Role of Syndecan-1 in Epithelial-Mesenchymal Transition and Malignant Transformation

During malignant transformation, cancer progression and metastasis normal epithelial cells undergo multiple orchestrated molecular and morphological changes leading to mesenchymal characteristics and migratory phenotype. One of the initial central steps in this epithelial-mesenchymal transition (EMT) [89, 90] is the transcriptional repression of epithelial markers, resulting in simultaneous loss of E-cadherin and syndecan-1 [91, 92]. Depletion of epithelial cells of cell surface syndecan-1 profoundly alters their morphology and anchorage-dependent growth [91], syndecan-1 thus being necessary to maintain the epithelial phenotype. Transforming growth factor-beta (TGF-*β*) can induce EMT in various cell types, and it appear to be responsible for the activation of a spectrum of EMT inducing transcription factors [90]. Among these SNAIL has been shown to repress the expression of syndecan-1 [93].

A coordinated loss of syndecan-1 and E-cadherin has been documented in many epithelial malignancies compared to their benign counterparts. Sequential loss or decreased expression of both these adhesion molecules was observed in skin associated with malignant transformation and their expression was further diminished with decreasing cell differentiation in invasive squamous cell carcinoma [94]. A significant reduction of both syndecan-1 and E-cadherin expression was also seen in severely dysplastic epithelium as compared to moderate dysplasia in colorectal-adenoma, with further reduction of both molecules in carcinomas compared to adenomas [33, 95]. In prostate syndecan-1 was expressed in basolateral surface of normal epithelium, changing to a granular cytoplasmic expression pattern in carcinomas [96], a switch in subcellular expression pattern linked to EMT.

## 5. Nuclear Localization of Syndecan-1

Though syndecan-1 is referred to as a membrane-bound protein, it has been detected in the nuclear compartment in different cell types [97, 98]. The presence and functions of heparan sulfate in the nucleus are a known phenomenon but the translocation of the core protein of the proteoglycan itself is relatively less studied. The concept of nuclear translocation of syndecan-1 was reinforced by the identification of the nuclear localization signals (NLS) at the cytoplasmic tail of the PG, explaining the mechanism of nuclear translocation. Syndecan-1 enters the cell membrane via raft dependent or receptor mediated endocytosis. The MKKK sequence is essential for the internalization by raft-dependent endocytosis [99] and the RMKKK motif is the minimal sequence required for its nuclear localization [100]. The complete route of syndecan-1 internalization is not yet elucidated, but it is known that the nuclear translocation of syndecan-1 is tubulin-dependent [97]. It has been shown that the full-length form of the syndecan-1 molecule (containing the ectodomain, transmembrane, and cytoplasmic domains) can translocate to the nucleus.

Recent studies show that the shed syndecan-1 also translocates to the nucleus of both tumor cells and bone-marrow-derived stromal cells [101]. The RMKKK sequence is found within the cytoplasmic domain of syndecan-1 and thus is not present in the shed molecule. This indicates that shed syndecan-1 enters the nucleus via an alternative mechanism. Exogenously added HS chains or the syndecan-1 ectodomain with its heparan sulfate chains could enter the nucleus as well [102]. For the nuclear translocation of the shed syndecan-1 sulfated HS chains of the PG and an unknown cargo (possibly a heparan sulfate-binding growth factor) bound to these heparan sulfate chains are required. This cargo remains bound to the shed syndecan-1 even after its translocation to the nucleus [85]. Its removal from shed syndecan-1 prevented the translocation to the nucleus, so it was hypothesized that this HS binding growth factor contains nuclear localization sequences [85].

The nuclear HS regulates gene expression by several mechanisms. First, it regulates the transcription machinery by inhibiting DNA topoisomerase, thereby preventing relaxation of DNA and the accessibility to transcription factors [103]. Moreover, HS inhibits transcription factors [104, 105] probably directly binding to them, as their DNA binding domain contains high affinity heparin binding sequences [106]. The nuclear HS can also regulate gene expression by modulating the acetylation status of histone proteins. Both nuclear syndecan-1 [101] and HS chains [102] inhibit nuclear histone acetyl-transferase activity and acetylation of histones thereby decreasing gene expression that drive tumor progression [101].

Transport of growth factors into the nucleus is another possible function of HS, as the heparin-binding growth factors and other macromolecules are internalized through HSPGs [100, 107–111]. This was shown in case of syndecan-1 for HGF [85] or FGF2 [107], with this latter colocalizing in the nucleus [100]. Other ligands, morphogens, peptides, and exosomes can also follow the same routes for entering the nucleus [110, 112, 113].

Nuclear HS has antiproliferative effects [106, 114, 115] and the extent of the growth inhibition depends on cell confluence, the composition, and sulfation grade of the nuclear HS [106] and it also varies in malignant and benign cells [116, 117]. Particularly, the highly sulfated HS chains present in the nucleus were shown to inhibit proliferation. Interestingly, with increasing cell confluence, the sulfation level also increases. In malignant mesothelioma cells, TGF*β* inhibited the nuclear translocation of syndecan-1 in parallel with an antiproliferative effect [118]. There seems to be correlation between nuclear HS and cell cycle progression also, though the exact mechanisms of action and the cause effect relationship are not established yet. The nuclear entry of HS depends on certain cell-cycle phases [97] and cell cycle progression is regulated by the amount of nuclear HS or HSPG [35, 97, 117, 119–121], but in contrary, mitotic cells loose nuclear HS [106] and induced cell-cycle arrest inhibits nuclear translocation [100].

## 6. Syndecan-1 in Cancer: Differentiation Marker with Prognostic Value

Several studies demonstrate that syndecan-1 expression in cancer is significantly correlated with tumor cell differentiation and prognosis. The basal syndecan-1 level and its cellular localization are however crucial for understanding the sequential changes involving malignant transformation, tumor progression, and advanced or disseminated cancer stages. Consequently cell-membrane bound, stromal, and soluble shed syndecan-1 seem to carry different, highly tissue specific information for individual tumor types that has to be viewed as contributing parts of a whole spectrum (Table 1).

Experimental overexpression of full length syndecan-1 enhances cell-ECM cohesion and restricts cell migration, whereas the loss of the syndecan-1 ectodomain from the cell surface increases the migratory capacity of tumor cells [122]. Similarly, overexpression of the full-length syndecan-1 enhances fibrosarcoma cell adhesion, while constructs lacking the ectodomain inhibit adhesion [121]. In a breast cancer cell line overexpression of wild type syndecan-1 increased cell proliferation, whereas overexpression of constitutively shed syndecan-1 had the opposite effect [86]. Presence of syndecan-1 is associated with favorable outcome in lung cancer and mesothelioma [123, 124], but it can also promote the growth of other tumor types [125, 126]. Moreover, different research groups found either tumor promoting or tumor inhibiting effects in the same tumor type, such as in colorectal cancer [127, 128] or prostate cancer [93, 125]. These seemingly contradictory data might partly be resolved by considering the localization of syndecan-1 in addition to its expression level.

The expression of* cell surface syndecan-1* in tumor tissue is context-specific. For instance, compared to normal epithelial cells, decreased syndecan-1 expression has been found during malignant transformation of prostate cancer [93], and reduced cell-membrane syndecan-1 immunoreactivity was observed in many epithelial malignancies connected to various stages of tumor progression.

Syndecan-1 present in* the stromal component* of different malignant tumors generally indicates poor prognosis through promotion of tumor cell invasion and development of metastasis [129, 130] and it also might stimulate the growth of epithelial cells [131, 132]. Also high level of* soluble syndecan-1* generally associates with poor prognosis and it correlates to tumor burden, cancer invasiveness, and risk for metastasis.

### 6.1. Tumors of the Lung and Pleura

In malignant mesothelioma, syndecan-1 levels are generally low. Presence of syndecan-1 is related to differentiation state of mesothelioma cells. It is mainly present in epithelial phenotype and in the epithelial component of biphasic mesotheliomas and it correlates with favorable prognosis [124].

Generally adenocarcinomas show higher cell surface and soluble syndecan-1 levels than mesotheliomas [135, 171, 172], the latter indicating worse prognosis. Considering this, syndecan-1 was proposed as a putative diagnostic marker in distinguishing mesotheliomas from metastatic adenocarcinomas. In squamous cell lung carcinoma low cell surface syndecan-1 expression is associated with unfavorable outcome [123] and the majority of NSCLC express varying syndecan-1 reactivity by immunohistochemistry of tumor tissue. High serum syndecan-1 levels associate with poor outcome in both NSCLC and SCLC [133, 134, 173, 174].

### 6.2. Head- and Neck Carcinoma

Decreased syndecan-1 expression in epithelial cells is associated with tumor aggressiveness and poor survival in squamous cell head and neck carcinoma [137–139, 142]. Thus, the level of the syndecan-1 expression can be a novel prognostic factor in head and neck cancers [140]. In squamous oral carcinoma stromal syndecan-1 inversely correlates with tumor grade and invasiveness [137]. In the serum of larynx and hypopharynx carcinoma patients the soluble syndecan-1 levels decrease after surgery and/or radiotherapy and the levels may increase at the time of tumor recurrence. The reason for this could be that a part of soluble syndecan-1 originates from the tumor tissue. The low syndecan-1 serum level in these tumors was predictive for favorable outcome [141]. Syndecan-1 is uncommonly expressed in nasopharyngeal carcinoma samples, but its expression correlates with advanced clinical stages and poor outcome [143].

### 6.3. Gastrointestinal Malignancies

The expression of syndecan-1 is induced in the stroma of gastric cancer, where its presence correlates with poor prognosis. Epithelial expression of syndecan-1 negatively correlates with lymph node metastasis [144] and associates with a longer survival, whereas stromal syndecan-1 expression associates with a shorter survival [145]. Low expression of syndecan-1 significantly correlates with the invasion and metastasis of gastric carcinoma [146].

Syndecan-1 shedding is increased in colorectal cancer [83] and the loss of epithelial syndecan-1 is associated with advanced clinical stage and poor prognosis [128, 148]. Selective expression of syndecan-1 in tumor-initiating cell lines suggests a role of syndecan-1 for cancer stem-cells [149]. On the other hand, there are studies showing that syndecan-1 immunopositivity is associated with tumor size [127].

Syndecan-1 is expressed in human normal liver [175] and the loss of syndecan-1 expression is a typical feature of hepatocellular carcinoma with high metastatic potential, where syndecan-1 expression is reduced both at mRNA and at protein levels [150].

Syndecan-1 expression is heterogenous and variable in intensity and distribution in intrahepatic cholangiocarcinoma. Higher level of syndecan-1 in neoplastic cells is associated with inhibition of invasiveness* in vitro*. Reduced expression of syndecan-1 is correlated with poor histological differentiation, lymph node metastasis, and poor prognosis after surgical resection [176].

In pancreatic cancer [151] increased levels of membrane syndecan-1 were found. In this tumor type stromal syndecan-1 expression is an independent prognostic marker, whereas epithelial syndecan-1 expression predicts better prognosis only in resectable tumors [177].

### 6.4. Breast Cancer

Breast cancer is associated with increased cell-membrane syndecan-1 [154]. Its expression is also induced in the stromal cells adjacent to the cancer, particularly in tumors exhibiting an aggressive phenotype [152]. The loss of epithelial syndecan-1 correlated with the syndecan-1 stromal expression and is found to be a significant poor prognostic factor [153]. Studies from an* in vitro* breast cancer model have also suggested that syndecan-1 directly participates in tumor cell spreading and adhesion [14]. Syndecan-1 expression is induced in the stroma of invasive breast carcinomas in some cases [155], whereas other studies linked an unfavorable prognosis in breast carcinoma patients with syndecan-1 in tumor cells but a better prognosis for those lacking syndecan-1 expression within the stroma. Furthermore, epithelial syndecan-1 expression was associated with negative ER status, whereas stromal syndecan-1 expression was associated with positive ER status [156]. A recent study showed that the proportion of syndecan-1 positive cells correlated with tumor grade better than the amounts demonstrated by immunohistochemistry, nuclear grade, and localization of syndecan-1. The estrogen and progesterone receptors both correlated negatively with syndecan-1 staining [157].

### 6.5. Urogenital Cancers

Syndecan-1 promotes the growth and invasive/metastatic potential of endometrial tumors. Upregulation of syndecan-1 in a xenograft model leads to the development of proliferative and invasive/metastatic phenotypes in endometrial cancer. The growth advantage conferred by syndecan-1 overexpression was accompanied by increased tumor angiogenesis. Syndecan-1 seems to be early in the signal cascades necessary for the onset of endometrial cancer progression [126]. Loss of epithelial syndecan-1 expression and induction of stromal syndecan-1 expression are associated with reduced survival in patients with endometrial cancer [160]. Increased syndecan-1 staining was a poor prognostic factor for survival also in ovarian cancer, where syndecan-1 was also present in the stromal compartment [159].

In prostate cancer the syndecan-1 level is correlated inversely with tumor grade [93]. In normal prostate tissue syndecan-1 is expressed mainly by epithelial cells while in tumors an overall increase of syndecan-1 expression was observed in the tumor stroma along with its disappearance from tumor epithelial cells [161]. In tumor initiating cells and mouse model in contrary, it was found that syndecan-1 immunopositivity is associated with recurrence and it has a role in maintaining tumor stem cells [125].

Syndecan-1 expression has a prognostic value also in bladder cancer. Surface expression of syndecan-1 was inversely correlated with tumor stage in primary nonmuscle-invasive bladder cancer [162], while high stromal syndecan-1 was associated with poor prognosis [163]. Syndecan-1 is also present in the stroma of urothelial cancer indicating a possible use as clinically important diagnostic marker [164].

### 6.6. Hematological and Other Malignancies

Syndecan-1 is the main diagnostic and prognostic marker of myeloma, and its importance for hematological malignancies was recently reviewed [178]. It is present in the nucleus of myeloma cells, and amount of nuclear syndecan-1 is reduced upon elevation of heparanase expression. Increased levels of shed syndecan-1 in serum correlate to tumor burden and poor outcome in multiple myeloma [165]. The levels of syndecan-1 were higher also in Hodgkin lymphoma patients than controls [179]. Positive expression of syndecan-1 was found in the plasma cells in B-CLL [180].

Expression of syndecan-1 was found in malignant glioma cells [181] and is highly overexpressed in dedifferentiated liposarcoma [170].

## 7. Conclusion

Taken together, the multitude and diversity of molecular functions related to syndecan-1 and its different localization highlights a complex tissue specific and development related expression pattern that is perturbed in many tumors. Several studies support the idea that there is a complementary feature in cell surface, soluble, stromal, and nuclear localization patterns but simultaneous detection of these parameters is very sparse. This gives a fragmentary description of the syndecan-1 expression and isolated expression levels have to be expanded to cover the whole spectrum of localizations in an effort to push further our understanding of the plethora of molecular events connected to syndecan-1.

## Figures and Tables

**Table 1:** Prognostic significance of syndecan-1 in relation to its cellular localization.

	Stromal	Soluble	Cell Surface	References
	syndecan-1	syndecan-1	syndecan-1	
Intrathoracic cancers				
Lung		Unfavorable	Favorable	[91, 123, 124, 133, 134]
Mesothelioma		Unfavorable	Favorable	[124, 135]

Skin cancers				
Basal cell carcinoma			Favorable	[136]
Squamous cell carcinoma (oral and cutaneous)	Favorable		Favorable	[94, 137]

Head and neck cancers				
Head and neck	Unfavorable		Favorable	[129, 138–140]
Laryngeal, hypopharynx		Unfavorable		[141]
Nasopharyngeal			Inconclusive^*∗*^	[142, 143]

Gastrointestinal cancers				
Gastric	Unfavorable		Favorable	[144–147]
Colorectal	Unfavorable		Inconclusive^*∗*^	[127, 128, 148, 149]
Hepatocellular			Favorable	[150]
Pancreatic			Unfavorable	[151]

Breast cancer				
Breast cancer	Inconclusive^*∗*^		Inconclusive^*∗*^	[131, 152–157]

Urogenital cancers				
Cervical			Favorable	[158]
Ovarial	Unfavorable		Unfavorable	[159]
Endometrial	Unfavorable		Inconclusive^*∗*^	[126, 160]
Prostate	Unfavorable		Inconclusive^*∗*^	[93, 125, 161]
Bladder	Unfavorable	Unfavorable	Favorable	[162, 163]
Urothelial	Unfavorable			[164]

Hematological malignancies				
Myeloma			Unfavorable	[165–168]
Hodgkin's lymphoma			Unfavorable	[169]

Other cancers				
Thyroid	Unfavorable		Unfavorable	[130]
Liposarcoma			Unfavorable	[170]

*∗* denotes inconclusive results, where different studies show opposite prognosis.
